# An advanced machine learning framework for predicting and experimentally validating Nb and Ta effects on the mechanical behavior of high-entropy alloys with reduced experimental dependency

**DOI:** 10.1038/s41598-026-45211-y

**Published:** 2026-05-06

**Authors:** Sandeep Jain, Ayan Bhowmik, Nokeun Park, Pradyumn Kumar Arya, Ankur Srivastava

**Affiliations:** 1 School of Materials Science and Engineering, Yeungnam University, Gyeongsan, 38541 Republic of Korea; 2 Department of Materials Science and Engineering, Indian Institute of Technology Delhi, Hauz Khas, New Delhi, 110016 India; 3 Department of Mechanical Engineering, Indian Institute of Technology Delhi, Hauz Khas, New Delhi, 110016 India; 4 Institute of Materials Technology, Yeungnam University, Gyeongsan, 38541 Republic of Korea; 5 Department of Mechanical Engineering, Manipal University Jaipur, Jaipur, 303007 India

**Keywords:** Machine learning, Mechanical behaviour, High entropy alloys, Refractory materials, Engineering, Materials science, Mathematics and computing

## Abstract

This study employs machine learning (ML) models such as Decision Tree (DT), Extra Tree (ET), K-Nearest Neighbors (KNN), Support Vector Regressor (SVR) and Lasso to predict how Ta and Nb content effects the mechanical behavior of the CoCrFeNi HEAs at diverse compositions. ET and KNN models developed as the most effective forecasters, accomplishing high R^2^ values of 0.985 and 0.984 respectively. The valuation of the mechanical behaviour with experimental data is very precise with the help of these models. The advanced methods like Hybrid Computational Variable Creation Method (HCVCM), Symbolic Variable Creation Method (SVCM), Data Structure Method (DSM) was further used which has enhance the prediction performance in terms of R^2^ value upto 0.991 for ET and 0.99 for KNN model. A novel stress-strain behaviour was produced for a new composition with novel Ta and Nb content, where ET and KNN demonstrated robust R^2^ values of 0.984 and 0.98, respectively. The strategy streamlines exploration of HEAs by cutting down extensive trial-and-error experiments, thereby conserving significant resources in terms of time, cost, and energy.

**Supplementary Information:**

The online version contains supplementary material available at 10.1038/s41598-026-45211-y.

## Introduction

 High-entropy alloys (HEAs) have increased noteworthy attention in recent years due to their unique microstructures and mechanical behavior^1–4^. These alloys are typically designed to form simple solid solution phases, primarily influenced by their four core effects^5–8^. The final phase constitution is strongly governed by various thermophysical parameters that are affected by the choice and proportion of alloying elements based on specific application needs^9–13^. HEAs strength offer a promising route for tailoring materials by optimizing the interplay between processing, microstructure, and mechanical performance^14–17^. Beyond this, multiphase HEAs are of great interest due to their complex microstructural configurations and mechanical stability^18–21^. Their applicability spans a wide array of sectors, including but not limited to automotive, aerospace, marine engineering, cryogenics, space technology, and nuclear power. The exceptional performance of HEAs can be attributed to their intricate compositions and unique structural traits.

This approach demonstrates effective through the formation of experimental alloy systems with FCC (Face Centered Cubic) and/or BCC (Body Centered Cubic) lattice structures, known for providing a promising combination of ductility and strength^22–24^. Nevertheless, optimizing the strength–ductility synergy in single-phase HEAs remains complex. FCC containing HEAs typically offer superior ductility but limited strength, while BCC containing HEAs with high strength are more prone to brittleness and reduced ductility. To overcome existing boundaries, investigators are discovering eutectic alloying strategies to design HEAs, particularly suited for refractory applications^25,26^. This involves modifying the composition by incorporating various alloying elements in different ratios to enhance the properties of single-phase HEAs^27,28^. For instance, Huan et al.^29^ studied the effect of hafnium (Hf) addition on the microstructure and mechanical behavior of CoCrFeNi alloys. Though, experimentally assessing all possible element combinations is both time-intensive and resource-demanding^30–32^. Such an approach is impractical for industries seeking rapid, energy-efficient solutions.

To meet industrial demands for quicker and more efficient HEA development, addressing current challenges is critical for resource efficiency and cost-effectiveness. Computational techniques including CALPHAD (Calculation of Phase Diagrams), molecular dynamics, and DFT (Density Function Theory) have been adopted extensively over the last ten years^33–36^. Nonetheless, these methods face persistent issues: CALPHAD is hindered by incomplete thermodynamic databases, DFT requires substantial computational resources, and overall, such approaches may not provide sufficiently adaptable or accurate models.

With the growing complexity of HEA systems, conventional design methods such as trial-and-error or traditional simulations are often inefficient. As a result, researchers are turning to machine learning, which has shown significant promise in accelerating alloy development^37–40^. Its strength lies in learning from data, adapting to new problems, and efficiently handling diverse inputs. When compared to older methods like CALPHAD or molecular dynamics, ML offers better scalability, improved automation, and broader problem-solving potential^41–45^.

Machine learning has become increasingly recognized for its ability to model mechanical properties with high accuracy, offering an alternative to traditional experimental methods^46–50^. Jain et al.^51^ has reported the design of eutectic high entropy alloys with the help of ML process to reduce the experimental dependency and to design the advanced materials efficiently. In the work reported by Elgack et al.^33^, the mechanical behavior of FeCoCrCuNi HEAs was examined through ML process. Yet, their framework was more focused on fitting known data rather than covering the new alloy system, thus limiting its generalization ability. While ML is showing inspiring results in property prediction and alloy optimization, its role in guiding the finding of new materials remains inadequately discovered.

Recent advances in materials informatics emphasize the use of data-driven models to establish direct composition–processing–property relationships, enabling accelerated development of structural materials^52,53^. Advanced variable generation frameworks like the Hybrid Computational Variable Creation Method (HCVCM), Symbolic Variable Creation Method (SVCM), Data Structure Method (DSM), and Robust Sparse Variable Creation Method (RSVCM) have been made possible by recent advancements in machine learning for materials and structural engineering^54^. By generating hybrid mathematical descriptors and carrying out structured feature selection, these methods improve predictive modeling and make it possible for machine learning models to more successfully capture nonlinear correlations between material properties. Shishegaran et al.^55^ employed several hybrid frameworks (HCVCM-SBSR, HCVCM-GEP, and HCVCM-ANFIS), to estimate concrete compressive strength using non-destructive testing parameters. Their results showed that the HCVCM-ANFIS model achieved the best predictive performance, improving the accuracy of ANFIS by enhancing key statistical indicators. Using these frameworks in applications involving concrete materials, recycled aggregates, reinforced structural elements, and other complicated engineering systems has significantly improved predicted accuracy, according to several studies^56,57^. This paradigm, strongly aligned with the objectives of the Materials Genome Initiative (MGI), promotes the integration of machine learning with targeted experiments to predict material performance prior to fabrication^58,59^. In this context, the present work focuses on the prediction of mechanical behavior, specifically the full stress–strain response of newly designed alloy compositions, rather than conventional phase or property regression using ML algorithms and advanced methods. By learning complex nonlinear relationships between composition and deformation behavior, the proposed framework enables rapid screening and validation of candidate alloys, thereby reducing experimental iterations and supporting performance-driven alloy design.

To address the present research gap, this study efforts on developing a ML based predictive framework that can estimate the mechanical behavior of novel alloy system, thereby lessening dependence on wide-ranging experimental trials. This approach significantly lowers the costs, time, and labor related with experimental methods. Motivated by these methodological advancements, the current study employs a similar feature-engineering approach that generates hybrid interaction descriptors and ratio-based variables from alloy compositional parameters, followed by sparse feature selection using LASSO. This allows the machine learning models to capture intricate nonlinear relationships governing the properties of CoCrFeNi high-entropy alloys. The central aim of the present work is to reduce experimental dependance by leveraging ML techniques. In particular, six ML models were employed to estimate the effect of Ta and Nb additions on the mechanical response of CoCrFeNi HEAs. These models were trained on existing experimental datasets to forecast stress-strain behavior for new compositions involving varying Ta and Nb content.

## Methodology

**Fig. 1:**
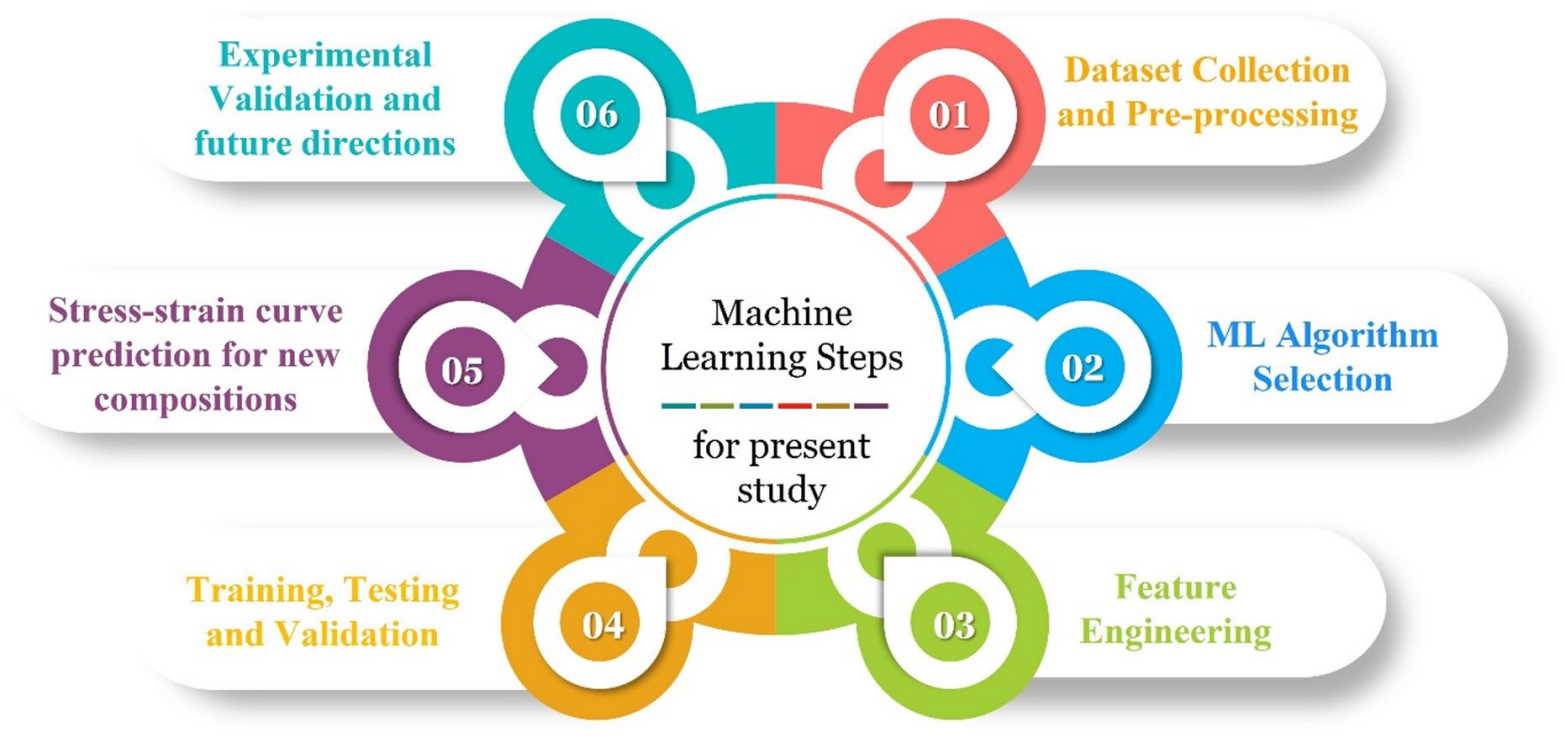
Complete ML-based workflow for stress-strain curve prediction and experimental validation in CoCrFeNi HEAs with Ta and Nb additions.

The complete flow chart of the present study describing all required steps of ML process is shown in Fig. 1.

### Collection of dataset

To carry out the present investigation, relevant datasets were sourced from various published studies^60,61^. The experimental outcomes from these studies providing mechanical property data for different alloy compositions, permitting a detailed analysis of how Ta and Nb influence mechanical behavior. A total of 880 data points were extracted from nine tensile stress–strain curves, comprising Ta (0.1, 0.2, 0.4, and 0.75 molar ratio) and Nb (0.25, 0.45, 0.75, and 1.2 molar ratio) containing CoCrFeNi alloys and the base CoCrFeNi alloy without Ta and Nb additions, with the number of data points varying depending on alloy composition. The mechanical testing was conducted at room temperature and at a strain rate of 10^− 3^ s^− 1^. Details of the compiled dataset are provided in Table 1. In this study, Ta content, Nb content and strain are treated as the input parameter and stress as the output. This strain-driven approach aligns with common practices in computational and machine learning-based materials modeling, where deformation is prescribed to predict material response. The input features were selected based on established physical metallurgy principles governing deformation behavior in high-entropy alloys. Composition-derived descriptors known to influence lattice distortion, solid-solution strengthening, and elastic–plastic response was used to ensure physical relevance to stress–strain behavior. Using strain as input allows systematic capture of the stress–strain curve, including both elastic and plastic regimes, and is directly relevant for engineering applications where strain limits are specified. The dataset includes significant compositional variation through controlled additions of Ta and Nb. This variation creates a diverse set of mechanical responses, enabling the model to learn generalized composition–property relationships. The consistent performance across the training, validation, and test sets further confirms that the dataset size sufficiently supports model generalization.

**Table 1:** Statistical Dataset description showing the selected input and output variables used in the present analysis.

S. no.	Parameters	Range of values	Minimum	Maximum	Mean	Standard deviation
1	Strain (%)	0–50	0	50	16.9	13.94
2	Ta content (molar ratio)	0–0.75	0	0.75	0.132	0.221
3	Nb content (molar ratio)	0–1.2	0	1.2	0.218	0.341
4	Stress (MPa)	0–2500	0	2500	747.36	668

### Data pre-processing

Before training the model, the dataset was carefully examined to detect missing values and unusual data points that might interfere with the learning process. It was ensured that the data contained no missing values, and any outliers were treated using statistical strategies like the standard deviation approach and interquartile range evaluation. We performed a box-plot analysis to examine missing values and outliers in the dataset before conducting the machine-learning study. The box plots are provided in Fig. S1 of the supplementary material. As seen in Fig. S1, the dataset contains no missing entries. The stress and strain data also show no outliers. For Ta and Nb concentrations, outliers appear at the 0.75% composition. Such variations are common in HEAs systems and reflect genuine compositional diversity rather than errors. Therefore, these points were not removed. Retaining them preserves the realistic chemical space of the alloy system and enhances the model’s ability to generalize and make accurate predictions.

### Selection of ML algorithms

The selection of appropriate ML algorithm is a crucial step to establish an optimization framework so that we can get reliable outputs and consistent results. This balance minimizes model complexity and helps avoid convergence to suboptimal solutions. Based on these considerations, in this study six different algorithms were selected based on their merits and demerits as given below:

To ensure a balanced evaluation of predictive performance, interpretability, and model complexity, six machine learning algorithms with complementary characteristics were selected. Extra Trees and CatBoost were employed as advanced ensemble methods capable of capturing complex nonlinear relationships and feature interactions within the dataset, with Extra Trees offering strong variance reduction through randomization and CatBoost providing robust gradient boosting performance with reduced overfitting. K-Nearest Neighbors (KNN) was included due to its simplicity and instance-based learning approach, which requires no assumptions about data distribution and allows exploration of local data behavior, although its effectiveness may decrease in high-dimensional spaces. The Decision Tree (DT) model was chosen for its transparent, rule-based structure, enabling intuitive interpretation of feature importance and decision pathways. Support Vector Regression (SVR) was applied to model nonlinear relationships using kernel functions that map the input variables into a higher-dimensional feature space. Finally, Lasso regression was adopted as a linear and highly interpretable baseline model with L1 regularization, facilitating feature selection and assessment of linear contributions while mitigating overfitting. Collectively, these models enable data-driven learning of the highly nonlinear stress–strain response, allowing the algorithms to capture elastic–plastic transitions and strain-hardening behavior directly from experimental descriptors without imposing predefined constitutive equations.

While the current study emphasizes forward prediction, the developed models provide a foundation for future integration with optimization-driven approaches to enable targeted composition design.

### Feature engineering

Heat maps play a significant role in machine learning analysis by offering an intuitive way to visualize feature distributions and their associations with labels. They enable the detection of correlations across features, helping to identify variables that may be redundant or strongly related, thereby affecting model outcomes. Often, these relationships are quantified using the Pearson correlation coefficient and presented in the heat map^62–64^.

Pearson correlation analysis was employed as an initial exploratory tool to quantify the strength and direction of pairwise linear associations among input variables. This step was used solely for preliminary feature understanding and visualization, not for establishing predictive relationships. The subsequent machine learning models inherently capture complex and nonlinear interactions between variables, ensuring that the overall framework is not limited by the linear assumptions of the Pearson metric.

**Fig. 2:**
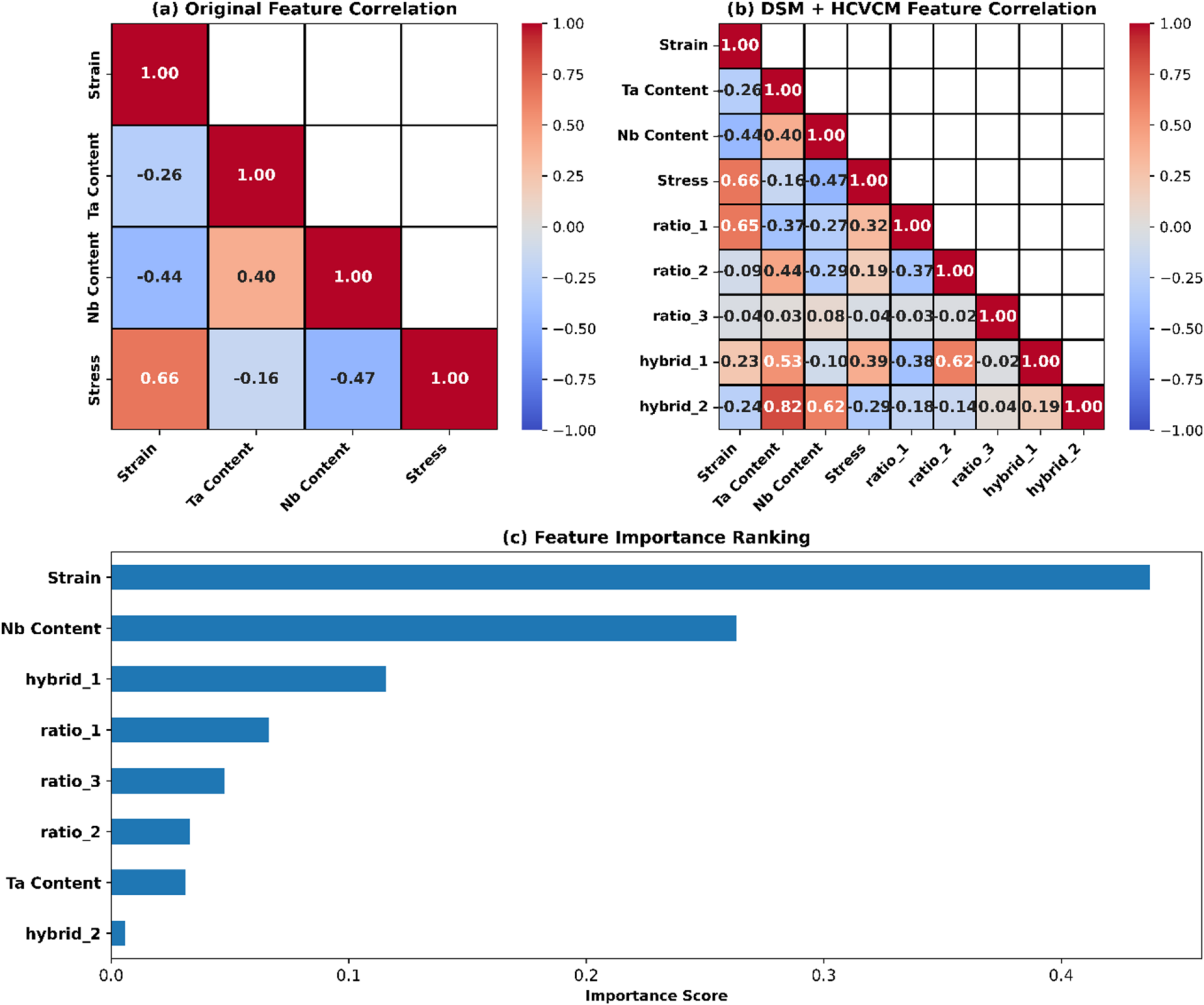
**a** The original descriptors’ correlation matrix. **b** Correlation matrix following the addition of engineered descriptors produced using DSM and HCVCM techniques, **c** feature importance ranking.

The correlation analysis and feature importance ranking for the descriptors utilized in the machine learning models are shown in Fig. 2. The initial descriptors have moderate correlations with the desired attribute (stress), as seen in Fig. 2a. Of these, strain exhibits the largest positive association (0.66) with stress, suggesting that deformation is the primary factor influencing the mechanical response. On the other hand, Ta content has a rather modest negative association (− 0.16) with stress, whereas Nb content shows a substantial negative connection (− 0.47).

As shown in Fig. 2b, new technical descriptors were added utilizing the DSM + HCVCM framework to improve the representation capabilities of the input variables. Ratio-based (ratio_1 = Strain/Ta content, ratio_2 = Ta content/Nb content, ratio_3 = Nb content/Strain) and hybrid interaction (hybrid_1 = Strain × Ta Content, hybrid_2 = Ta Content × Nb Content) features that are obtained from the elemental compositions and strain are among these descriptors. These developed features capture extra nonlinear interactions between the variables and the desired attribute, according to the correlation matrix. For instance, hybrid_1 and ratio_1 show clear relationships with stress, indicating that the alloy system’s mechanical behavior is influenced by a combination of compositional interactions. Additionally, strain has the highest important score (~ 0.43) in the prediction model, confirming its dominant influence, according to the feature importance analysis displayed in Fig. 2c. Nb content (~ 0.26) and hybrid_1 (~ 0.12) following that, showing that the prediction accuracy is highly influenced by both primary descriptors and designed interaction features. Ta content and hybrid_2, on the other hand, have comparatively lesser relevance, indicating a weaker direct contribution to the model predictions.

Overall, the findings show that using DSM + HCVCM tailored descriptors enhances the machine learning model’s ability to capture the structure–property correlations in the CoCrFeNi-based alloy system by better representing complicated compositional interactions.

### Hyperparameters optimization

The performance and stability of machine learning models heavily depend on effective hyperparameter optimization. A widely adopted approach for this is grid search, which systematically explores multiple parameter settings to determine the optimal choice^65–67^. A predefined range of parameters was evaluated through grid search, with five-fold cross-validation employed to select the most suitable configuration for reliable model performance. Based on this optimization, the algorithms were used to estimate the mechanical properties corresponding to varying Ta and Nb concentrations, with the chosen hyperparameters presented in supplementary data Table S1.

### ML process

This dataset content input parameters such as Strain values, different Ta & Nb contents and output parameters such as Stress values. This dataset was divided into three parts in the proportion of 80:10:10 ratio for training, testing and validation subsets respectively. An extra sensitivity analysis was carried out utilizing various training–testing ratios, such as 80–20, 75–25, 70–30, and 65–35, in order to assess the robustness of the chosen data partitioning technique. The same feature scaling, preprocessing, and 5-fold cross-validation hyperparameter optimization technique were used for every setup. The coefficient of determination (R²), root mean square error (RMSE), and mean absolute error (MAE) were used to assess the model’s performance. This analysis allows assessment of the stability of the proposed machine learning framework under different data partitioning conditions.

The analysis employed six machine learning techniques, and the two with the highest predictive accuracy were selected. These models were used to estimate the mechanical characteristics of HEAs with varying Ta and Nb content, highlighting the influence of these elements. The predictions were then cross verified with experimental measurements, ensuring greater dependability of the model for practical use. The performance of the machine learning models was evaluated using the coefficient of determination (R²), root mean square error (RMSE), and mean absolute error (MAE). RMSE and MAE were calculated in their dimensional form (MPa) to preserve the physical meaning of the stress prediction error. In addition, normalized RMSE and MAE values were computed by dividing the dimensional errors by the mean experimental stress and expressed as percentages (%), enabling a relative comparison of prediction accuracy across different alloy compositions.

A 5-fold cross-validation (CV) technique in conjunction with hyperparameter optimization was used to guarantee robustness and avoid overfitting. Five subsets of the training dataset were generated, four of which were utilized for model training and one of which was used for validation each cycle. In order for each subset to serve as the validation fold once, this procedure was carried out five times. The mean ± standard deviation of the assessment metrics across the five folds was used to determine each model’s cross-validation performance. The standard deviation measures the model’s stability with regard to data partitioning, whilst the mean value indicates the average predictive performance. The cross-validation performance of all models has been added in the supplementary file in Table S2.

Following the identification of the optimum hyperparameters, the final model was trained on the complete training dataset and then assessed on an external validation dataset that was entirely separate from the training, cross-validation, and model tuning processes. An objective evaluation of the model’s capacity for generalization is guaranteed by this independent validation.

## Results and discussion

### ML models

**Fig. 3:**
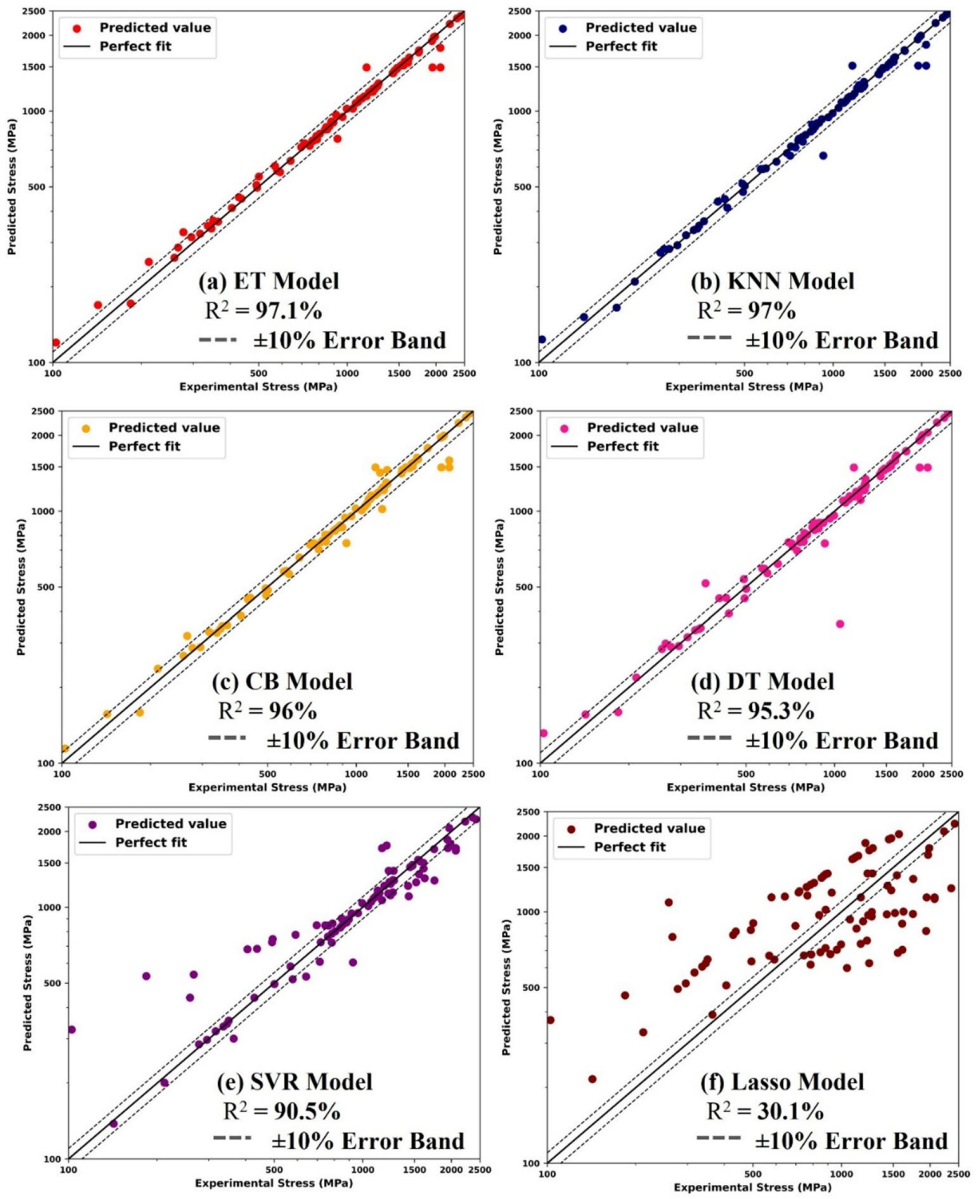
Comparison of testing results of six different regression models. **a** ET, **b** KNN, **c** CB, **d** DT, **e** SVR, **f** Lasso.

**Fig. 4:**
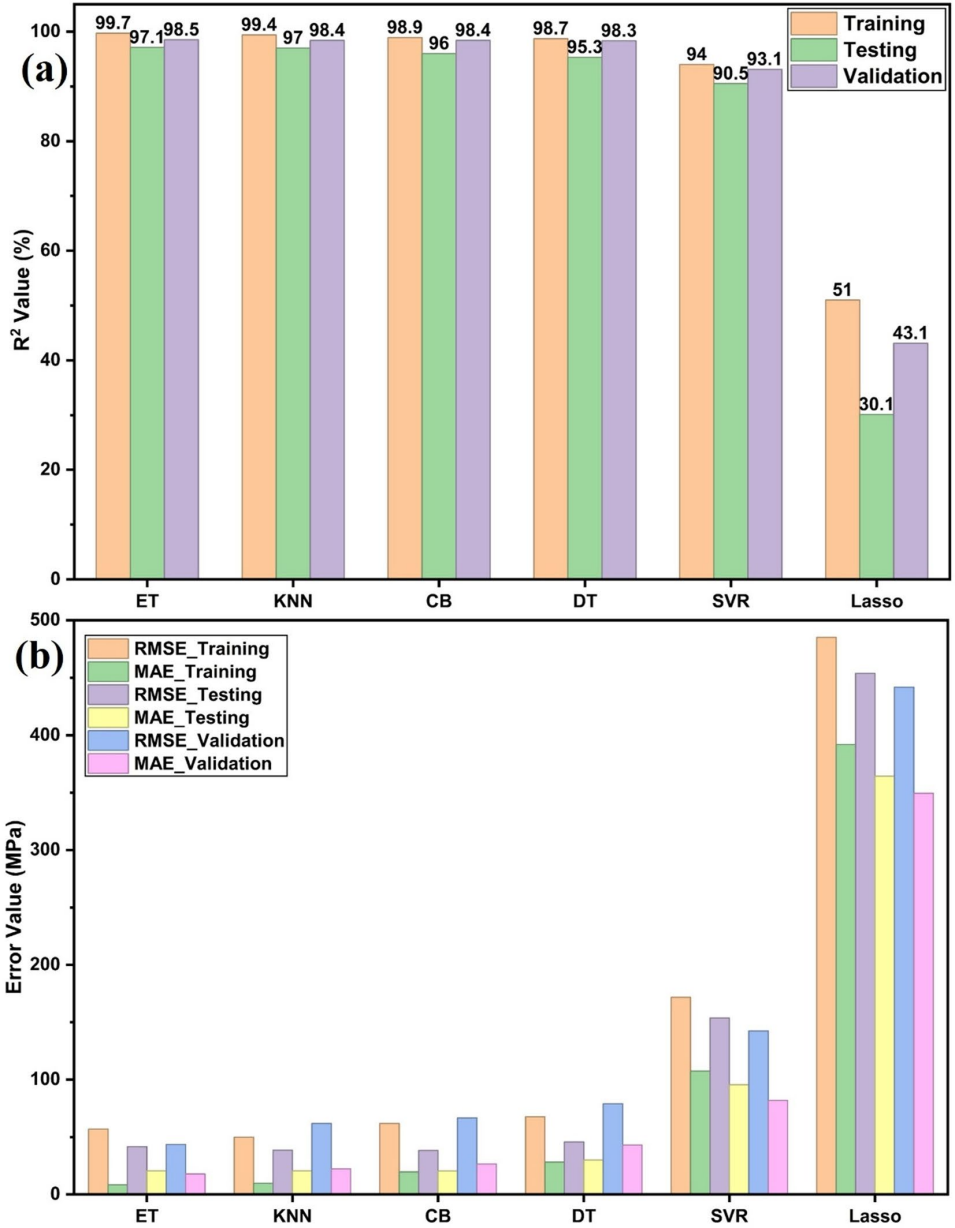
Performance matrices of six different ML models used during different steps of analysis. **a** R^2^ Values, **b** RMSE and MAE values.

To predict the mechanical behaviour of HEAs, the first step is to train the algorithms to understand the relationship between input and output parameters. Using optimized hyperparameters, six separate algorithms were trained and subsequently tested. Figure 3 displays and compares the results obtained from the training and testing stages for each algorithm. Figure 3 shows that initial four models achieving very good relationship in testing and showing very close alignment with the expected outcomes while other models are showing deviation. For better understanding these results, evaluation matrices during these two phases have been calculated and shown in Fig. 4 and these matrices also revealed that initial four models are showing good performance. Both dimensional (MPa) and normalized (%) RMSE and MAE values are reported to provide complementary insight into the absolute and relative prediction errors of the models. These performances are shown in Table S3 to S5 in the supplementary file.

**Fig. 5:**
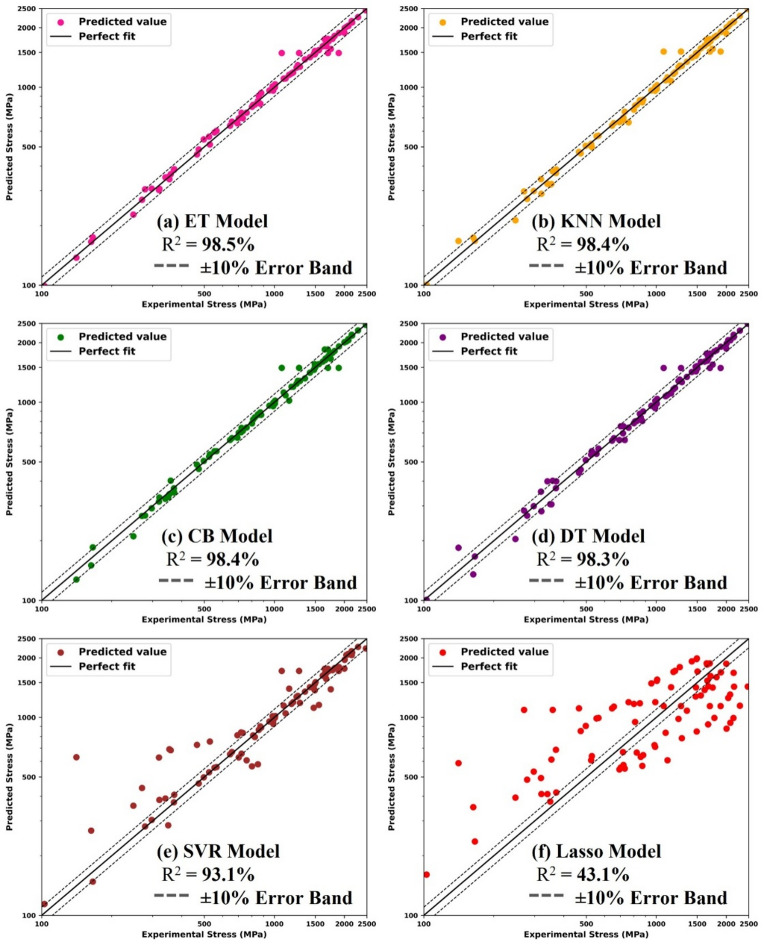
Comparison of validation results of six different regression models. **a** ET, **b** KNN, **c** CB, **d** DT, **e** SVR, **f** Lasso.

To examine these models, one more step is required and that is validation step. The validation step is completed by using the validation dataset which was kept aside and not included in training and testing. By doing so we can evaluate the performance of these models in better way with more accuracy and reliability. The results from the validation step for each model are shown in Fig. 5. The evaluation matrices of validation step are also shown in Fig. 4. Figures 4 and 5 show that initial 4 models are showing good performance and first two models (ET and KNN) achieved superior performance among them. These models demonstrate strong predictive capability for mechanical behavior under different Ta and Nb levels, highlighting their applicability in forecasting the performance of novel alloy compositions.

### Sensitivity analysis of training-testing ratio

**Fig. 6:**
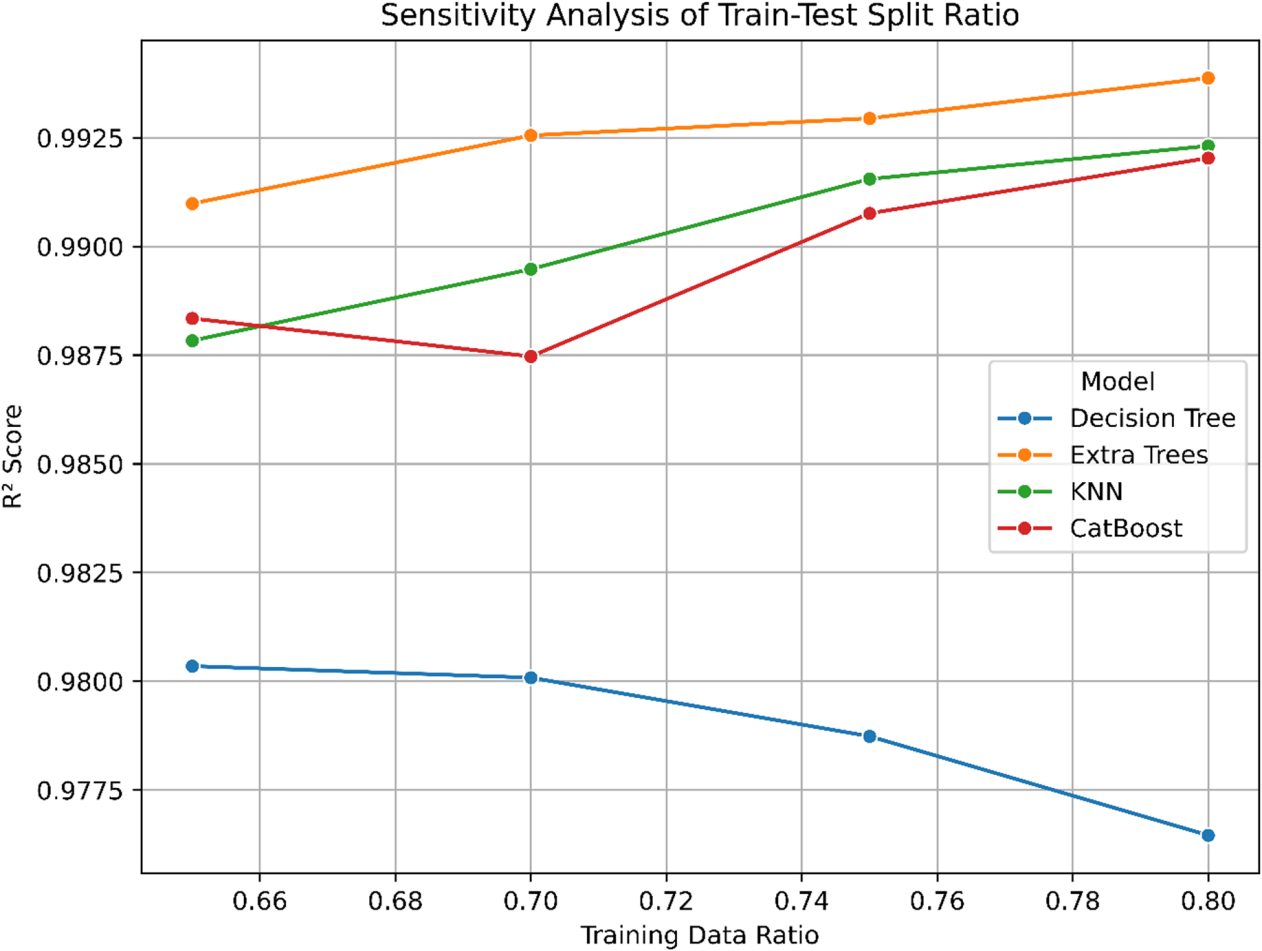
Sensitivity analysis of train test split ratio.

Multiple training–testing ratios (80–20, 75–25, 70–30, and 65–35) were used in a sensitivity analysis to investigate the impact of data partitioning on model performance. Table S6 and Fig. 6 provides a summary of the findings. The findings show that the models’ prediction performance is very consistent across various partitioning techniques. With R^2^ values ranging from 0.9909 to 0.9939, the Extra Trees model in particular consistently produced the best prediction accuracy. For every split that was assessed, the KNN and CatBoost models continued to perform well, with R^2^ values greater than 0.987. Additionally, the Decision Tree model demonstrated consistent predictive power with R^2^ values ranging from 0.976 to 0.980.

The overall predictive performance remained consistently strong, despite minor increases in RMSE and MAE as the test proportion grew. These results show that the machine learning models that have been constructed are resilient and do not exhibit considerable sensitivity to moderate changes in the training-testing ratio. As a result, the initial 80% training fraction guarantees adequate data availability for model training while offering dependable performance.

### Comparison of predictive performance of top models

Analysis of the training, testing, and validation results shows that the top four models exhibit closely comparable performance. To assess whether their predictive abilities differ significantly, a paired t-test was conducted using the mean absolute error (MAE) of each model. The statistical outcomes reveal that ET, KNN, and CatBoost deliver similar performance levels (ET vs. KNN: t = 0.690, *p* = 0.492; ET vs. CatBoost: t = 0.395, *p* = 0.694). In contrast, DT performs significantly worse than ET (t = − 4.037, *p* < 0.001).

To complement the statistical analysis, kernel density estimation (KDE) curves of the prediction errors for all models were plotted in Fig. S2 (provided in the supplementary file). The KDE distributions for ET, KNN, and CatBoost nearly overlap and show lower error regions, whereas DT displays a distinctly broader error distribution, reinforcing the t test findings.

Overall, ET, KNN, and CatBoost emerge as the most reliable models in this study. However, to reduce computational cost during further analysis, we selected only the top two models among these three, based on their R² values. Although a single model could have been used, retaining two models allows for comparative evaluation and a clearer understanding of predictive robustness. The final selection of one model can then be made based on the concluding results.

### Computational efficiency and practical deployment considerations of all models

Training time, prediction time, latency, throughput, and model size were used to assess the models’ computing efficiency in addition to predictive accuracy (Table S2 to S4) and shown in Table 2. With a compact model (~ 30.9 KB), low latency (~ 0.0075 ms/sample), and a very quick training time (0.017 s), KNN demonstrated an exceptional balance between accuracy and efficiency, making it appropriate for high-throughput alloy screening. Both Extra Trees (ET) and CatBoost (CB) produced a big model (~ 9.6 MB), suggesting their suitability for offline, high-accuracy design jobs, but they also needed greater training costs. With somewhat less generality, Decision Trees (DT) allowed for the fastest inference and maximum throughput, facilitating real-time prediction applications. Despite being computationally easier, SVR and Lasso had lower prediction reliability because they were unable to adequately capture the nonlinear interactions. Overall, the comparison shows that when maximal predicted precision is needed, ET/CB are better, while KNN offers the best trade-off between speed and accuracy. Therefore, ET and KNN models are chosen for new prediction work.

**Table 2:** Computational efficiency of all used models.

S. no.	Model	Training time (s)	Prediction time test (s)	Latency (ms/sample)	Throughput (samples/s)	Model size (KB)
1	ET	0.52	0.0072	0.0821	1.22e + 04	9589.73
2	KNN	0.017	0.0006	0.0075	1.32e + 05	30.9
3	CB	4.26	0.0009	0.0109	9.10e + 04	222.95
4	DT	0.224	0.0001	0.0013	7.72e + 05	96.42
5	SVR	0.073	0.0066	0.0758	1.32e + 04	25.71
6	Lasso	2.048	0.00007	0.0008	1.15e + 06	0.522

### Comparison of model performance with the addition of advanced methods

Many advanced methods like DSM, HCVCM etc. was used to enhance the predictability of used ML models. The results of validation performance by using ML models without and with incorporating advanced methods are shown in Table 3 for all models. It is clear from Table 3 that validation performance of ML models has been enhanced in a great manner. For instance, the validation R^2^ increase from 98.5% to 99.1% for Extra Trees, from 98.4% to 99% for KNN, from 98.4% to 98.8% for CatBoost, and from 98.3% to 98.9% for Decision Tree. While SVR showed a minor decline (93.1% to 89.4%), the LASSO model shown a significant improvement from 43.1% to 67.6%. These findings suggest that most models’ predictive power can be enhanced by hybrid feature creation.

**Table 3:** Validation performance by using ML models without and with incorporating advanced methods.

S. no.	Model	Without advanced methods		With advanced methods	
		*R*^2^ (%)	RMSE (MPa)	*R*^2^ (%)	RMSE (MPa)
1	ET	98.5	43.5	99.1	61.5
2	KNN	98.4	61.8	99	63
3	CB	98.4	66.7	98.8	68.6
4	DT	98.3	79	98.9	65.7
5	SVR	93.1	142.4	89.4	204.8
6	Lasso	43.1	441.9	67.6	357.5

### Prediction and experimental validation the effect of new Ta content on HEAs

**Fig. 7:**
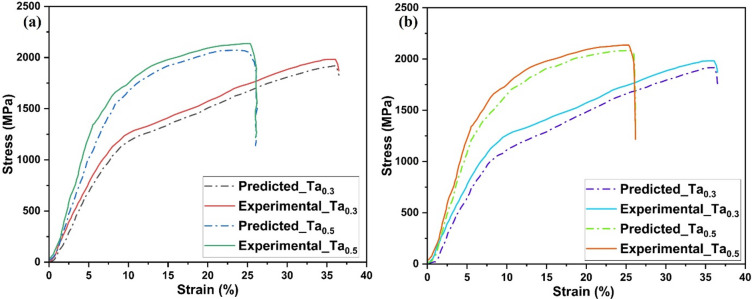
Experimental validation and comparison between actual and predicted stress-strain curve using **a** ET, **b** KNN models for the prediction for new Ta content.

In order to save time, reduce costs, and minimize experimental effort, developing a machine learning approach for predicting flow stress behavior over various conditions is necessary. This would eliminate the need for extensive physical tests, providing a more accurate and efficient means. Specifically, the mechanical behavior for new composition by adding Ta as 0.3 and 0.5 molar ratio was predicted with the aid of the two best-performing models across all strain values. Figure 7 confirms the accuracy of the proposed model by showcasing a close match between predicted and experimental flow stresses. This alignment supports the model’s potential to streamline the materials development process by minimizing the reliance on numerous physical experiments.

### Prediction and experimental validation the effect of new Nb content on HEAs

**Fig. 8:**
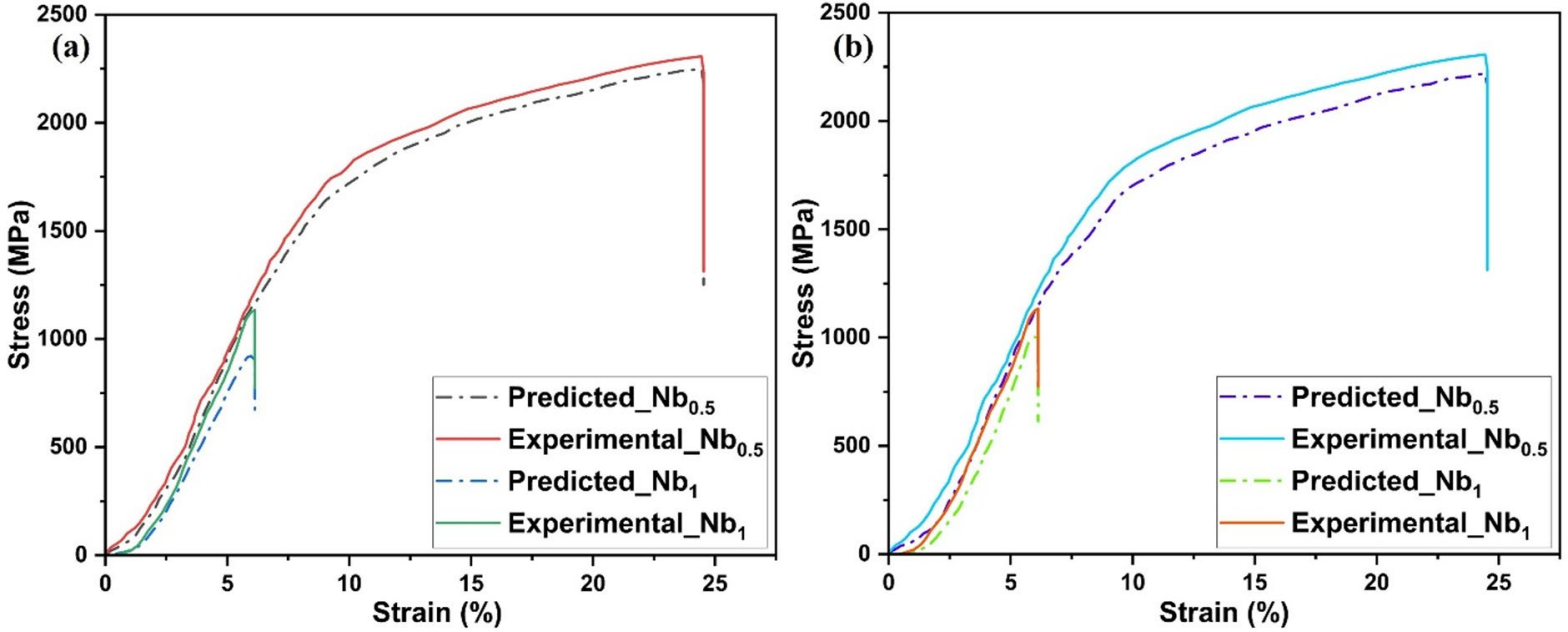
Experimental validation and Comparison between actual and predicted stress-strain curve using **a** ET, **b** KNN models for the prediction for new Nb content.

Additionally, the mechanical behavior for new composition by adding Nb as 0.5 and 1 molar ratio was predicted with the aid of the two best-performing models across all strain values. The predicted flow curves at new Nb content are shown in Fig. 8 which exhibit behavior comparable to the experimental results after comparison with predicted values. The comparison between experimental and predicted flow curves, revealing that both ET and KNN models closely matched the experimental values.

The slight underestimation of stress observed at higher strain levels (Figs. 6, 7) is primarily attributed to the limited number of training samples available in the high-strain regime, where deformation becomes strongly nonlinear. Since the dataset is more densely populated at lower and intermediate strains, the model is biased toward learning the dominant trends, leading to conservative predictions at the extremes. Additionally, microstructural mechanisms such as strain hardening and lattice distortion introduce sharp stress evolution that is difficult to fully resolve without denser experimental representation. This behavior reflects data distribution constraints rather than a limitation in the predictive framework.

### Prediction performance for new composition

**Fig. 9:**
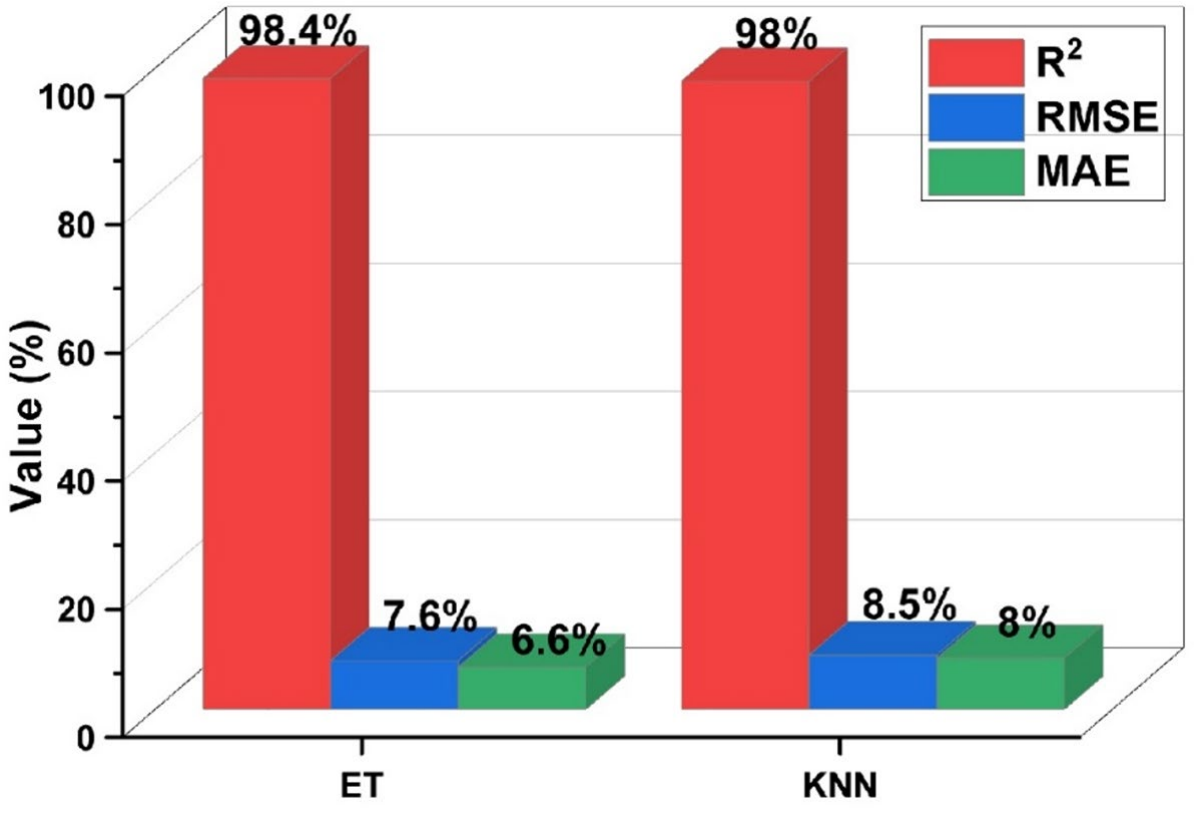
Performance of prediction of mechanical behaviour at new Ta and Nb content using ET and KNN models.

The prediction performance of these two models for the prediction of mechanical behaviour for new Ta and Nb content are shown with the help of evaluation matrices. These evaluation matrices are shown in Fig. 9. The ET model surpassed KNN, achieving an R^2^ of 98.4%, Mean Absolute Error (MAE) of 6.6%, and Root mean Square Error (RMSE) of 7.6%, while KNN achieved an R^2^ of 98%, MAE of 8%, and RMSE of 8.5%.

The strong predictive performance of the Extra Trees (ET) and K-Nearest Neighbors (KNN) models arises from their ability to capture the inherently nonlinear and locally varying interactions present in multicomponent alloy systems. ET, as a randomized ensemble method, models complex relationships between compositional and thermodynamic descriptors while reducing variance through decorrelated tree construction, making it well suited for high-dimensional materials data. KNN, a non-parametric approach, learns localized trends in composition space by leveraging similarities among alloys with comparable descriptors. In contrast, linear models such as Lasso may oversimplify these relationships, while SVR and single decision trees can be more sensitive to parameter selection and overfitting. Overall, these results indicate that flexible, data-driven approaches are more effective for representing the complex structure–property interactions in the studied alloy system.

Despite obtaining high R^2^ values, the possibility for overfitting was thoroughly investigated. The models did not memorize the training data, as evidenced by the cross-validation findings, which demonstrated stable prediction performance with low variance across folds. Additionally, the excellent generalization power of the suggested framework is shown by the close agreement between training and validation outcomes.

This ML model successfully showing the mechanical behaviour with a smaller number of experiments that would require multiple experimental iterations. This strategy demonstrates the capability of optimized ML model to generalize it for new compositions and reducing the need for extensive testing and accelerating alloy design for this system. Although the present study focuses on widely adopted machine learning algorithms with rigorous validation, recently developed advanced learning frameworks may be explored in future studies to further enhance predictive capability.

## Conclusion

The present study leverages different ML models to examine the role of Ta and Nb content in determining the mechanical characteristics of CoCrFeNi HEAs. Important results from this analysis are highlighted below:

- Six ML methods have been effectively employed to forecast the mechanical behavior of alloys with varying compositions, including different amounts of Ta and Nb.
- Among the tested models, ET and KNN stood out with superior validation metrics, including R² scores of 0.985 and 0.984, RMSEs of 7% and 6.9%, and MAEs of 3.2% and 3%. The ET model provided highly accurate predictions, showing close agreement with the experimental data.
- Four new stress-strain curves were generated for a new composition by adding Ta 0.3 and 0.5 molar ratio and Nb 0.5 & 1 molar ration using predictions from the ET and KNN models.
- These innovative predictions exhibited excellent performance, achieving R² values of 0.984 and 0.98, along with RMSE of 7.6% and 8.5%, and MAE of 6.6% and 8% for the ET and KNN approaches, respectively.
- Furthermore, inspired by advanced frameworks such as HCVCM, SVCM, DSM, and RSVCM, additional ratio-based and hybrid descriptors were introduced to capture nonlinear interactions among strain, Ta, and Nb contents. The correlation and feature importance analyses confirmed that these engineered variables improved the predictive capability and robustness of the machine learning models. This demonstrates the effectiveness of interaction-aware descriptor engineering for modeling complex composition–property relationships in high-entropy alloys while reducing experimental dependency.
- This forecasting approach marks a significant advancement in analyzing the mechanical properties of HEAs, minimizing experimental reliance, providing reliable prediction of mechanical behaviour for new compositions and saving considerable resources. The ability to capture mechanical trends with fewer experiments represents the primary novelty of this work and provides a practical pathway for rapid materials development.
- The presented framework provides a scalable strategy for data-driven mechanical property prediction that can be readily adapted to other HEA families through system-specific retraining.
- Although the present study focuses on data-driven prediction of the stress–strain behavior, future work will incorporate uncertainty quantification techniques, such as ensemble learning or Bayesian approaches, to evaluate prediction confidence. In addition, the integration of physically based constitutive relations into the machine learning framework (e.g., physics-informed modeling) will be explored to enforce governing laws and improve model generalization and reliability.

## Supplementary Information

Below is the link to the electronic supplementary material.

Supplementary Material 1

## Data Availability

Upon request, the data will be provided.
