# Supplementary material for: An advanced machine learning framework for predicting and experimentally validating Nb and Ta effects on the mechanical behavior of high-entropy alloys with reduced experimental dependency
Source: Sci Rep. 2026 May 6;16:20866. doi: 10.1038/s41598-026-45211-y (PMC13338202; doi:10.1038/s41598-026-45211-y)
Supplement: Supplementary file 1 — Supplementary Material 1 [file 41598_2026_45211_MOESM1_ESM.docx]

**An Advanced Machine Learning Framework for Predicting and Experimentally Validating Nb and Ta Effects on the Mechanical Behavior of High-Entropy Alloys with Reduced Experimental Dependency**

Sandeep Jain^a, b#^, Ayan Bhowmik^b^, Nokeun Park *^a,d#^,* Pradyumn Kumar Arya ^c^, Ankur Srivastava ^e #^

*^a^School of Materials Science and Engineering, Yeungnam University, Gyeongsan, 38541,*

*Republic of Korea*

*^b^Department of Materials Science and Engineering, Indian Institute of Technology Delhi, Hauz Khas, New Delhi, 110016, India*

*^c^ Department of Mechanical Engineering, Indian Institute of Technology Delhi, Hauz Khas, New Delhi, 110016, India*

*^d^Institute of Materials Technology, Yeungnam University, Gyeongsan,38541, Republic of Korea*

*^e^ Department of Mechanical Engineering, Manipal University Jaipur, Jaipur, 303007, India*

# Corresponding Authors: [ankur.srivastava@jaipur.manipal.edu](mailto:ankur.srivastava@jaipur.manipal.edu), [sandeepmbm20@gmail.com](mailto:sandeepmbm20@gmail.com), [nokeun_park@yu.ac.kr](mailto:nokeun_park@yu.ac.kr)

**Table S1.** Optimized Hyperparameters for used models

| **S. No.** | **Models** | **Hyperparameters Search Space** | **Best Hyperparameters** |
| --- | --- | --- | --- |
| 1 | ET | {bootstrap: [True, False],  max_depth: [5, 10, 15, 20],  min_samples_leaf: [1, 2, 4],  min_samples_split: [2, 5, 10],  n_estimators: [50, 100, 200, 300]} | {bootstrap: False,  max_depth: 20,  min_samples_leaf: 1,  min_samples_split: 2,  n_estimators: 300} |
| 2 | KNN | {algorithm: ['auto', 'ball_tree', 'kd_tree', 'brute'],  leaf_size: [20, 30, 40],  n_neighbors: [3, 5, 7, 10, 15],  p: [1, 2],  weights: ['uniform', 'distance']} | {algorithm: brute,  leaf_size: 20,  n_neighbors: 3,  p: 2,  weights: distance} |
| 3 | CatBoost | {depth: [4, 6, 8, 10],  iterations: [50, 100, 200, 300],  l2_leaf_reg: [1, 3, 5, 7],  learning_rate: [0.01, 0.05, 0.1, 0.3]} | {depth: 10,  iterations: 300,  l2_leaf_reg: 1,  learning_rate: 0.1} |
| 4 | DT | {max_depth: [3, 5, 7, 10, 15],  max_features: ['auto', 'sqrt', 'log2'],  min_samples_leaf: [1, 2, 4],  min_samples_split: [2, 5, 10]} | {max_depth: 15,  max_features: sqrt,  min_samples_leaf: 1,  min_samples_split: 2} |
| 5 | SVR | {C: [0.1, 1, 10, 100],  degree: [2, 3, 4],  epsilon: [0.1, 0.2, 0.5],  gamma: [0.1, 1],  kernel: ['rbf', 'linear', 'poly', 'sigmoid']} | {C: 100,  degree: 2,  epsilon: 0.1,  gamma: 1,  kernel: rbf} |
| 6 | Lasso | {alpha: [0.0001, 0.001, 0.01, 0.1, 1.0, 10.0],  fit_intercept: [True, False],  max_iter: [1000, 2000],  selection: ['cyclic', 'random']} | {alpha: 0.0001,  fit_intercept: True,  max_iter: 2000,  selection: random} |


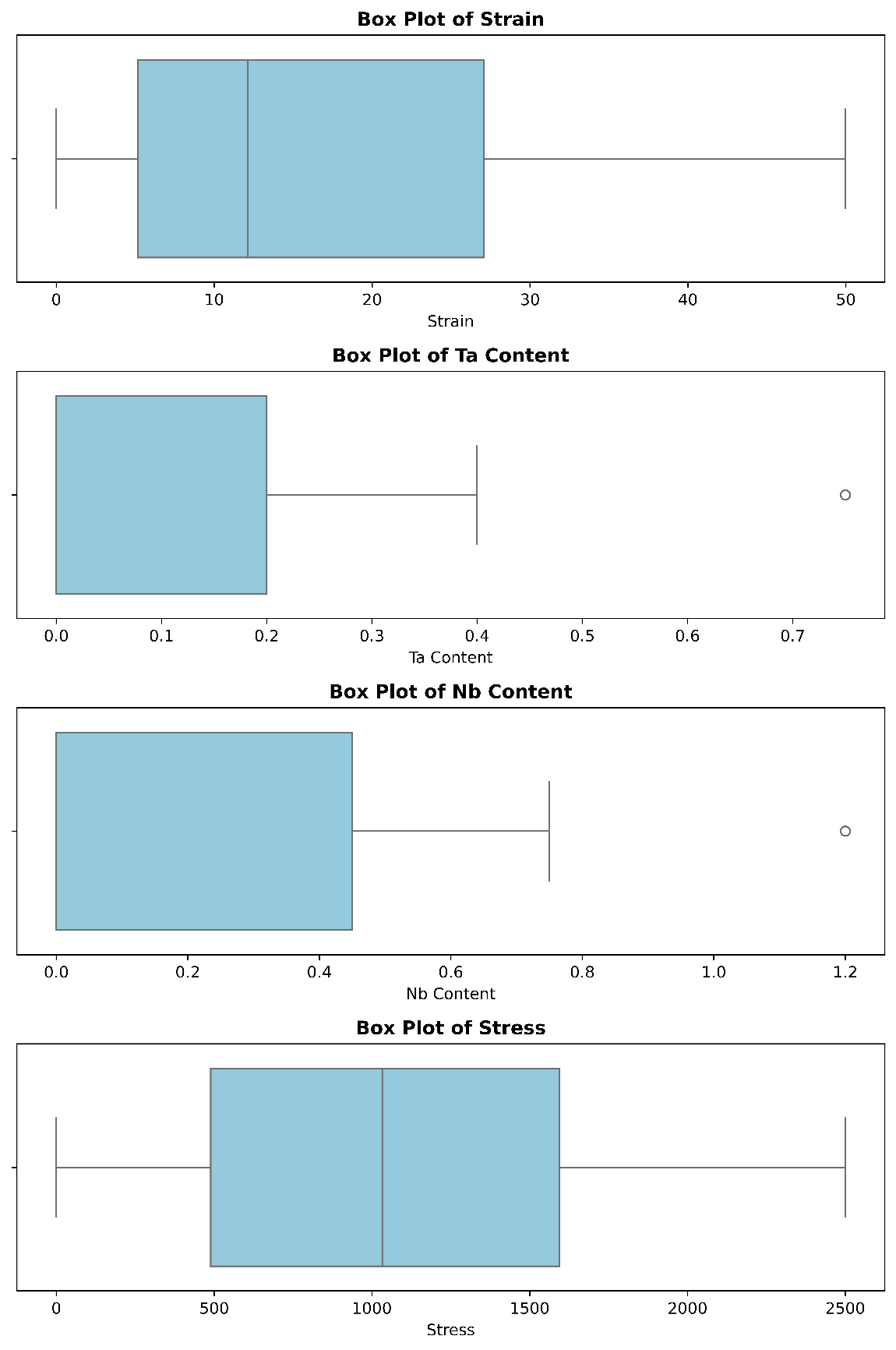


**Fig. S1** Box plots for input and output parameters for showing outliers and missing data in used dataset


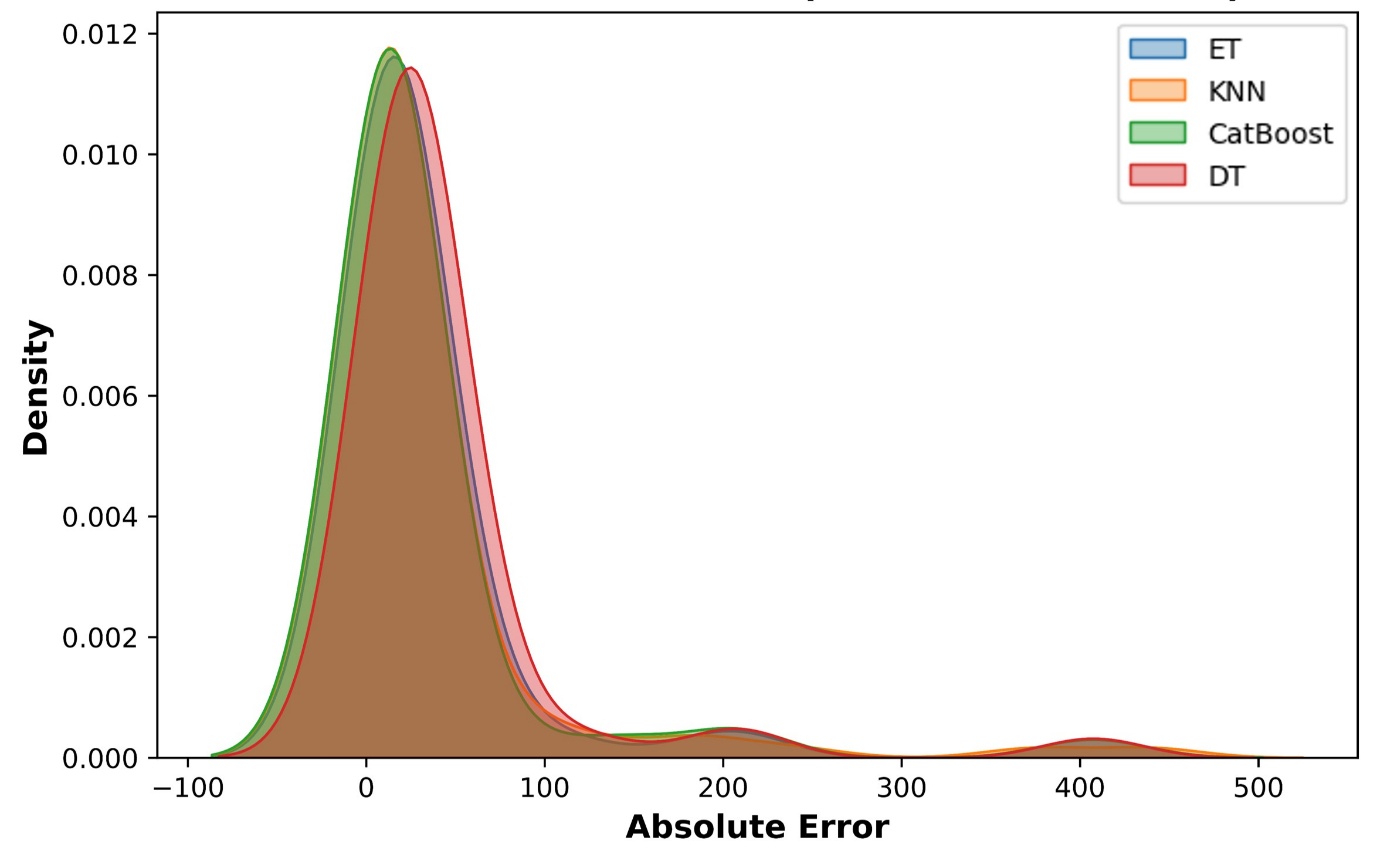


**Fig. S2** Kernel density estimation (KDE) plots of the prediction errors for top four models

**Table.S2** Comparison of all model’s Cross validation performance with mean and standard deviation value

| **S.No.** | **Model** | **CV Mean R^2^ (%)** |
| --- | --- | --- |
| 1 | ET | 98.9 ± 0.385 |
| 2 | KNN | 98.6 ± 0.421 |
| 3 | CB | 98.8 ± 0.4 |
| 4 | DT | 97.9 ± 0.65 |
| 5 | SVR | 92.5 ± 1.2 |
| 6 | Lasso | 46.3 ± 7.63 |

**Table.S3** Comparison of all model’s performance during training in terms of percentage (%) and MPa unit

| **S.No.** | **Model** | **R^2^** | | **RMSE** | | **MAE** | |
| --- | --- | --- | --- | --- | --- | --- | --- |
|  |  | **In %** | **In MPa** | **In %** | **In MPa** | **In %** | **In MPa** |
| 1 | ET | 99.7 | 99.3 | 3.6 | 56.95 | 0.5 | 8.5 |
| 2 | KNN | 99.4 | 99.1 | 3.7 | 49.76 | 0.5 | 9.6 |
| 3 | CB | 98.9 | 98.8 | 5.1 | 61.8 | 1.8 | 19.6 |
| 4 | DT | 98.7 | 98.1 | 3.6 | 67.7 | 0.5 | 28.2 |
| 5 | SVR | 94 | 93.5 | 15.7 | 171.8 | 9.6 | 107.6 |
| 6 | Lasso | 51 | 47.8 | 45.1 | 485.3 | 36 | 391.9 |

**Table.S4** Comparison of all model’s performance during testing in terms of percentage (%) and MPa unit

| **S.No.** | **Model** | **R^2^** | | **RMSE** | | **MAE** | |
| --- | --- | --- | --- | --- | --- | --- | --- |
|  |  | **In %** | **In MPa** | **In %** | **In MPa** | **In %** | **In MPa** |
| 1 | ET | 97.1 | 99.6 | 9.1 | 41.5 | 3.3 | 20.5 |
| 2 | KNN | 97 | 99.4 | 9 | 38.5 | 3.2 | 20.5 |
| 3 | CB | 96 | 99.5 | 10.6 | 38.3 | 4.3 | 20.4 |
| 4 | DT | 95.3 | 98.8 | 11.5 | 45.8 | 4.6 | 30 |
| 5 | SVR | 90.5 | 94.7 | 16.3 | 153.7 | 10.4 | 95.5 |
| 6 | Lasso | 30.1 | 53.7 | 44.4 | 453.8 | 37.6 | 364.3 |

**Table.S5** Comparison of all model’s performance during validation in terms of percentage (%) and MPa unit

| **S.No.** | **Model** | **R^2^** | | **RMSE** | | **MAE** | |
| --- | --- | --- | --- | --- | --- | --- | --- |
|  |  | **In %** | **In MPa** | **In %** | **In MPa** | **In %** | **In MPa** |
| 1 | ET | 98.5 | 99.5 | 7 | 43.5 | 3 | 17.9 |
| 2 | KNN | 98.4 | 99 | 7.2 | 61.8 | 3.1 | 22.3 |
| 3 | CB | 98.4 | 98.8 | 7.7 | 66.7 | 3.8 | 26.5 |
| 4 | DT | 98.3 | 98.4 | 8.6 | 79 | 4.9 | 43 |
| 5 | SVR | 93.1 | 94.8 | 15 | 142.4 | 9 | 82 |
| 6 | Lasso | 43.1 | 50.5 | 43.4 | 441.9 | 34.8 | 349.5 |
